# Machine Learning-Based Anomaly Detection in NFV: A Comprehensive Survey

**DOI:** 10.3390/s23115340

**Published:** 2023-06-05

**Authors:** Sehar Zehra, Ummay Faseeha, Hassan Jamil Syed, Fahad Samad, Ashraf Osman Ibrahim, Anas W. Abulfaraj, Wamda Nagmeldin

**Affiliations:** 1 FAST School of Computing, National University of Computer and Emerging Sciences, Karachi 75030, Pakistan or sehar.ifti@gmail.com (S.Z.); or ummay.faseeha@juw.edu.pk (U.F.);; 2 College Education & Literacy Department, Khursheed Government Girls Degree College, Government of Sindh, Karachi 75230, Pakistan; 3 Department of Computer Science, Main Campus, Jinnah University For Women, Karachi 74600, Pakistan; 4 Faculty of Computing & Informatics, Universiti Malaysia Sabah, Jalan UMS, Kota Kinabalu 88400, Sabah, Malaysia; 5 Cyber Security Research Lab, Faculty of Computing and Informatics, Universiti Malaysia Sabah, Jalan UMS, Kota Kinabalu 88400, Sabah, Malaysia; 6 Creative Advanced Machine Intelligence Research Centre, Faculty of Computing and Informatics, Universiti Malaysia Sabah, Jalan UMS, Kota Kinabalu 88400, Sabah, Malaysia; 7 Department of Information Systems, King Abdulaziz University, Rabigh 21911, Saudi Arabia; awabulfaraj@kau.edu.sa; 8 Department of Information Systems, College of Computer Engineering and Sciences, Prince Sattam bin Abdulaziz University, Al-Kharj 11942, Saudi Arabia; w.nagmaldin@psau.edu.sa

**Keywords:** network function virtualization (NFV), Internet of Things (IoT), security challenges, anomaly detection, cyber-attacks, machine learning based, supervised learning, unsupervised learning

## Abstract

Network function virtualization (NFV) is a rapidly growing technology that enables the virtualization of traditional network hardware components, offering benefits such as cost reduction, increased flexibility, and efficient resource utilization. Moreover, NFV plays a crucial role in sensor and IoT networks by ensuring optimal resource usage and effective network management. However, adopting NFV in these networks also brings security challenges that must promptly and effectively address. This survey paper focuses on exploring the security challenges associated with NFV. It proposes the utilization of anomaly detection techniques as a means to mitigate the potential risks of cyber attacks. The research evaluates the strengths and weaknesses of various machine learning-based algorithms for detecting network-based anomalies in NFV networks. By providing insights into the most efficient algorithm for timely and effective anomaly detection in NFV networks, this study aims to assist network administrators and security professionals in enhancing the security of NFV deployments, thus safeguarding the integrity and performance of sensors and IoT systems.

## 1. Introduction

**Figure 1:**
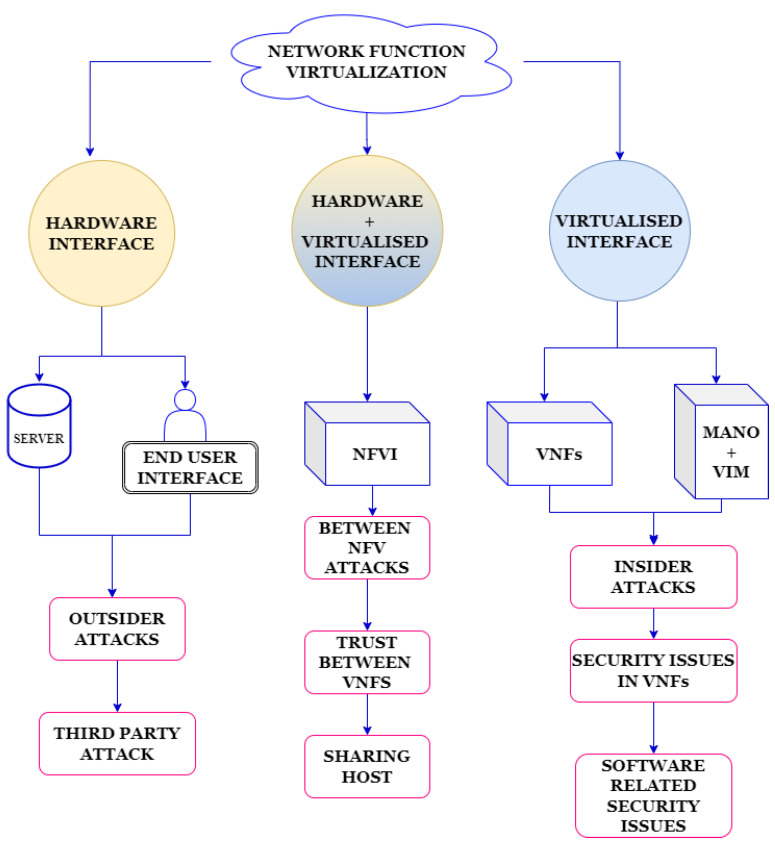
NFV infrastructure.

The industry has adopted the virtualization of network elements in recent years. Network virtualization offers many benefits, including easier implementation and management of network resources and services, potentially reducing operating costs and spurring innovation [1]. Network virtualization moves network connectivity and operations from dedicated hardware to software that runs on virtual machines or containers [2]. Implementing network function virtualization (NFV) offers several advantages, such as enhanced agility, flexibility, security, and scalability, as well as reduced hardware costs and power consumption of the network [3]. Network function virtualization (NFV) is also of great importance in the context of sensor and IoT networks. With the rapid growth of IoT devices and the increasing demand for diverse network functionalities, NFV offers significant advantages [4]. By virtualizing network functions and decoupling them from dedicated hardware, NFV enables the flexible deployment and management of network services in resource-constrained IoT environments [5]. The ability to dynamically allocate and scale virtualized network functions allows for the efficient utilization of limited resources and adaptability to changing IoT network requirements. NFV also enhances the security and reliability of sensor and IoT networks by enabling the deployment of virtualized security functions, such as firewalls and intrusion detection systems [6]. Overall, NFV empowers IoT network operators to optimize resource usage, improve network flexibility, and enhance security, making it a crucial technology for efficiently operating sensor and IoT networks [7]. The NFV architecture consists of three primary functional blocks, as Figure 1 illustrates.

Network function virtualization infrastructure (NFVI): This component encompasses the necessary hardware infrastructure to facilitate the deployment of NFV. It includes servers, storage units, and network resources that house virtual network functions (VNFs). Additionally, it provides the essential computation, storage, and networking capabilities required for network resource virtualization and dynamic allocation [8].

NFV management and orchestration function (MANO): MANO plays a pivotal role in managing and orchestrating the NFV environment. It consists of three subcomponents: NFV orchestrator (NFVO), virtualized infrastructure manager (VIM), and virtual network function manager (VNFM). The NFVO oversees the overall organization and coordination of VNFs and resources within the NFV infrastructure. The VIM manages virtualized infrastructure resources, including virtual machines’ allocation, monitoring, and lifecycle management. The VNFM is responsible for managing the lifecycle of individual VNF instances, including their deployment, scaling, and termination [9].

Virtual network function services block (VNFs): This block encompasses a variety of virtual network functions (VNFs) that deliver specific network functionalities. VNFs are software-based implementations of traditional network functions, such as firewalls, routers, load balancers, and intrusion detection systems. These VNFs can be combined to create flexible and customizable network services through dynamic deployment, scaling, and chaining. The NFV services block empowers network operators to efficiently deliver and manage a wide range of network services in a more agile and cost-effective manner [9].

Each of these functional blocks plays a critical role in the successful deployment and operation of NFV. Together, they collaborate to virtualize network functions, manage resources, and provide flexible network services, revolutionizing the construction and operation of networks.

Virtualization has not only brought numerous benefits but has also introduced new security risks and vulnerabilities, creating opportunities for cyber attacks [10]. Within the NFV architecture, three types of attacks are possible, as depicted in Figure 1.

Firstly, inside attacks occur when an attacker takes advantage of vulnerabilities related to software validation and configuration [11]. These vulnerabilities are represented in part ‘c’ of Figure 1. Secondly, outside or third-party attacks target the vulnerabilities of the hardware infrastructure and the network’s perimeter. The areas susceptible to these attacks are illustrated in part ‘a’ of Figure 1. Lastly, attacks between virtual network functions (VNFs) can occur due to the sharing of resources. Attackers exploit vulnerabilities within shared resources among VNF services to carry out malicious activities, as shown in part ‘b’ of Figure 1. To ensure the integrity and reliability of the network infrastructure, organizations implementing NFV must be aware of these security risks. It is crucial for them to take appropriate measures to mitigate this risks [9]. By implementing robust security measures and continuously monitoring the NFV environment, organizations can minimize the potential for cyber attacks.

Various effective techniques and methods based on network security have been proposed by the researchers that also include anomaly detection [12]. Anomaly detection identifies and addresses performance and security-related issues associated with anomalies by analyzing patterns, behaviors, and observations that deviate significantly from the norm [13]. By detecting an anomaly before it affects the quality of service and security of the NFV, timely countermeasures can be taken [14]. It can also identify vulnerabilities and hidden threats in the NFV infrastructure by monitoring packets, network traffic, performance data, and network protocols and can also determine network-based intervention [15]. Network-based anomaly detection in NFV involves analyzing different kinds of data to detect abnormal behavior in virtualized network functions. The data types that may be processed include network traffic, system-level, application-level, and security-related data [16]. Advanced research in anomaly detection in NFV has focused on developing new techniques and algorithms to effectively process these data [17]. These techniques may involve machine learning, data mining, or other advanced analytics methods and may be applied to real-time or historical data. Moreover, specialized tools and platforms have been developed to collect, process, and analyze data in NFV environments, such as OpenStack Monasca and the OPNFV Doctor project [18]. This paper surveyed state-of-the-art anomaly detection techniques in NFV networks, covering various aspects, such as approaches, classification, causes, use cases, and limitations of anomaly detection in NFV [19].

In this paper, we surveyed state-of-the-art anomaly detection in the NFV network. To the best of our knowledge, few studies have surveyed network-based anomaly detection using machine learning techniques. For instance, Pang, Guansong, et al. [20] (2021), Nassif, Ali Bou, et al. [21] (2021), Wang, Song, et al. [22](2021), Gebremariam, A. A., Usman, M. and Qaraqe, M. [23] (2019), Alam, Iqbal, et al. [24] (2020), Ghaffar, Zeba, et al. [25] (2021), Lohrasbinasab, Iraj, et al. [26] (2022), Shah, Ali Hussain, et al. [27] (2022), Gallego-Madrid, Jorge, et al. [28] (2022), Ahmed, Md, et al. [29] (2021), Nunez-Agurto, Daniel, et al. [30] (2022), and Di Mauro, Mario, et al. [31] (2021) have surveyed different aspects of anomaly detection in NFV network but they lack in considering all aspects of anomaly detection in the NFV network using machine learning techniques.A brief comparison of our survey paper with all these existing survey papers is shown in Table 1.

Ref. [20] provides a comprehensive survey of deep learning techniques for anomaly detection. The paper follows a structured approach and provides detailed information on the advantages and limitations of deep learning for anomaly detection. However, the paper does not critically assess the surveyed techniques and does not cover other anomaly detection techniques beyond deep learning. The paper acknowledges the technical difficulty of implementing deep learning techniques but provides a performance comparison of the surveyed techniques. Ref. [21] provides comprehensive coverage of machine learning techniques and anomaly detection, and it uses a thorough and systematic approach to the literature. The study identifies gaps and future research, and presents its findings in a clear and well-organized manner, making it a valuable resource for researchers and practitioners. However, the paper has several weaknesses, including limited discussion on practical implementation issues, real-world applications, and the quality of selected studies. Additionally, there is no evaluation or comparison of the selected studies and limited discussion on the limitations of the study. The survey by Wang, Song, et al. [22] provides a structured approach and comprehensive coverage of machine learning techniques in network anomaly detection. However, the paper has some weaknesses, such as the limited focus on specific types of network anomaly detection, a lack of clarity in some sections, and limited discussion on future research directions. Additionally, the paper has limited coverage of other techniques and limited discussion on implementation challenges. The survey by Gebremariam, A. A., Usman, M. and Qaraqe, M. [23] comprehensively covers a range of topics related to the application of AI and machine learning in software-defined networking (SDN) and network function virtualization (NFV). The authors use a structured approach to present the advantages and limitations of various AI and machine learning techniques in this domain. However, the paper does not cover other relevant techniques in the area of SDN and NFV, and there is limited discussion on the technical difficulties that may arise during the implementation of these techniques. Additionally, there is no performance comparison of the different approaches discussed.The paper by Alam, Iqbal, et al. [24] provides a comprehensive survey of network virtualization techniques for the Internet of Things (IoT) using software-defined networking (SDN) and network function virtualization (NFV). The paper is well structured, provides a critical assessment of the surveyed techniques, and discusses their advantages and limitations. However, the paper lacks a comparative analysis of the surveyed techniques, and some important techniques are not covered. Additionally, the paper may be technically challenging for readers without a background in SDN, NFV, or IoT. Ref. [25] provides a comprehensive review of machine learning, software-defined networking, and IoT applications, including their advantages and limitations, and offers critical insights into the current state of the art. While the paper follows a structured approach and covers a wide range of topics, it mainly focuses on these three areas and does not cover other related techniques. The paper may be challenging for readers who are not familiar with the technical aspects of these topics and does not provide a performance comparison between different techniques and applications. Lohrasbinasab, Iraj, et al. [26] provide a structured approach to network traffic prediction, covering statistical and machine learning-based techniques. The paper offers insights into the advantages and limitations of different approaches and provides a critical assessment of the current state of the art. However, the paper does not cover other related techniques and may be technically challenging for readers without a background in network traffic prediction. Additionally, the paper does not provide a performance comparison between different techniques, which could be useful for readers who want to compare them. Shah, Ali Hussain, et al. [27] propose an approach for automated log analysis and anomaly detection using machine learning. Their paper follows a structured approach and presents the advantages and limitations of the proposed methodology. The paper provides a critical assessment of the proposed approach, pointing out its limitations and future directions for improvement. While it covers other techniques in log analysis and anomaly detection, it is not comprehensive, and may be challenging for readers without a background in machine learning. The paper provides a comparison of the proposed approach with other methods, but it could be more comprehensive. Ref. [28] follows a structured approach and presents the advantages and limitations of the proposed approach, with a critical assessment of the methodology. It covers other techniques in log analysis and anomaly detection but not in depth. The technical difficulty of the paper may be high for readers without a background in machine learning. While the paper provides a comparison of the proposed approach with other methods, the comparison could be more comprehensive. Ref. [29] is a detailed analysis of different IDS techniques in SDNs. It provides a comprehensive overview of the current state of research in this field, discussing the advantages, limitations, and critical assessment of the different techniques. The paper follows a structured approach and includes a performance comparison of the different techniques. However, it could include more recent research, detailed descriptions of the techniques, examples, and solutions to the challenges associated with implementing IDS techniques in SDNs. The paper also lacks coverage of other techniques, such as rule-based systems and anomaly detection. Nunez-Agurto, Daniel, et al. [30] provides a comprehensive review of machine learning techniques for traffic classification in software-defined networking. However, the paper has some weaknesses, including a lack of coverage of other applications of machine learning in networking, a need for more detailed description of the methodology used in the review, and more analysis on the limitations and potential biases of the studies reviewed. The paper assumes a high level of technical knowledge in the field of networking and machine learning and could benefit from a more detailed comparison of the performance of the different machine learning techniques reviewed. Di Mauro, Mario, et al. [31] provide a comprehensive review of supervised feature selection techniques in network intrusion detection. The authors use a structured approach to divide the techniques into different categories based on their underlying algorithms and discuss their advantages and limitations, making the paper suitable for readers with technical expertise. The paper also covers a wide range of supervised feature selection techniques and compares their performance, which can help readers make informed decisions about which technique to use. However, the authors could have provided more critical assessment and insights into the limitations of the techniques. Our survey paper provides a comprehensive and structured approach to anomaly detection in NFV networks using machine learning. It addresses the gaps and limitations of existing studies by covering a wide range of features, such as the advantages and limitations of different techniques, critical assessment of existing solutions, coverage of other techniques, and technical difficulty. Additionally, we conducted a performance comparison that demonstrates the efficacy of different proposed approaches in detecting malicious anomalies in VNF networks. Our study provides practical guidance and valuable insights to network administrators and security professionals for selecting the most suitable machine learning-based technique for anomaly detection in NFV networks.

Furthermore, our survey paper comprehensively reviews the current state-of-the-art machine learning-based anomaly detection techniques in the NFV network. We propose a thematic taxonomy to categorize the existing literature and analyze the reviewed papers based on this taxonomy, providing a comprehensive insight into the current research trends and identifying the open research issues and challenges in anomaly detection for NFV networks. This makes our survey paper a valuable resource for guiding future research in this field.

In addition, our survey paper goes beyond the scope of previous studies by covering other cutting-edge techniques, such as hybrid approaches, incremental learning, transfer learning, ensemble methods, and explainable AI. We also provide a design framework that can guide practitioners in implementing machine learning-based anomaly detection in NFV networks. These additions make our survey paper more comprehensive and valuable, as it offers a complete overview of the latest developments in this field.

With these additional features, our survey paper provides a more comprehensive and up-to-date view of the state-of-the-art in anomaly detection for NFV networks, making a significant contribution to the field of anomaly detection in NFV networks. As such, it can serve as a valuable resource for researchers and practitioners alike.

In the next section, we cover all these aspects of NFV anomaly detection. Additionally, this paper analyzes a few research techniques proposed to efficiently find an anomaly in NFV [32]. The sections of the paper are arranged as follows: Section 2 presents a taxonomy of network-based anomaly detection in NFV. Section 3 elaborates on state-of-the-art different anomaly detection mechanisms and gives their comparison. Section 4 reveals research issues and challenges. Section 5 concludes the whole paper and discusses future work.

## 2. Taxonomy of Network-Based Anomaly Detection in NFV

**Figure 2:**
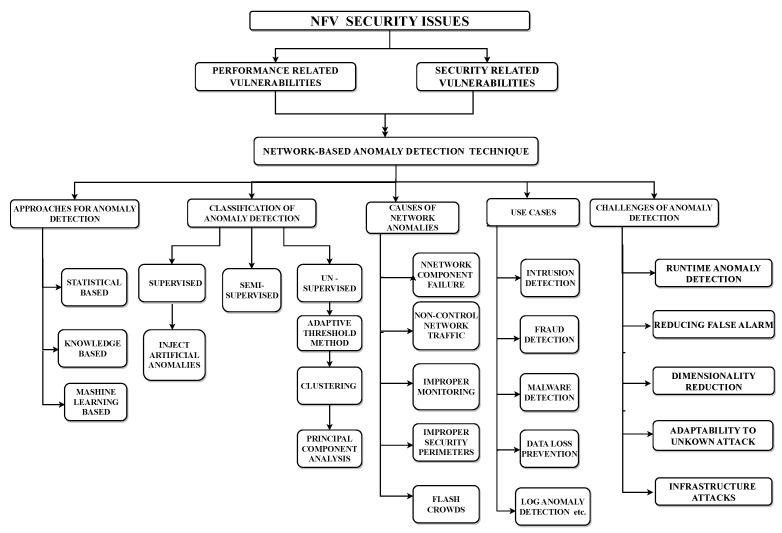
Thematic taxonomy of network-based anomaly detection in NFV.

In this section, we discuss the thematic taxonomy of anomaly detection in NFV. Figure 2 highlights some of the important features that help us propose a better mechanism for finding anomalies in the NFV network.

### 2.1. NFV Security Issues

NFV works in a virtualized environment that has various vulnerabilities. We categorized it into two main types, performance-related vulnerabilities and security-related vulnerabilities [33]. Performance-related vulnerabilities occur due to weakness in the network architecture, lack of data flow control and backup devices, the poor configuration of software and security devices, etc. [34] which will affect the performance of the NFV network, and attackers will exploit these vulnerabilities for attacks [35]. Security-related threats, including malicious attacks, are more easily encountered in NFV because NFV is a shared resource architecture, primarily when implemented on a cloud platform. In addition to third-party interference, the use of public networks for communication also makes NFV security more vulnerable than traditional hardware networks [36].

### 2.2. Network-Based Anomaly Technique

Anomaly detection techniques are used to identify the abnormal behavior of the overall network and identify not only active and passive attacks but also dynamic and novel malicious attacks [37]. Anomaly detection techniques have some advantages over firewalls or other malware tools, as they can identify abnormal behavior across hosts, networks, and distributed levels. This paper specifically focuses on network-based anomaly detection techniques in NFV networks [38].

Network anomaly detection involves monitoring traffic, analyzing various metrics, and using techniques such as statistical analysis, machine learning, and rule-based methods to detect anomalous behavior [39]. In an NFV network, network functions can be dynamically deployed, scaled, and migrated, making it difficult to detect anomalies. Therefore, specialized techniques and tools are needed, such as distributed monitoring and analysis, and techniques that focus on detecting anomalies in the behavior of virtualized network functions themselves [40]. Network anomaly detection in NFV is a specialized form of anomaly detection that focuses on identifying anomalies within virtualized network functions in an NFV environment [41].

#### 2.2.1. Approaches for Anomaly Detection

Anomaly detection has different approaches to finding anomalies in the network, but three of them are more commonly implemented, that is, statistical-based, knowledge-based, and machine learning-based approaches [42]. In statistical-based anomaly detection, abnormalities related to network data traffic are identified using statistical measures, e.g., [43] the mean, standard deviation, uni-variant, and multi-variant. There are several efficient statistical methods for analyzing the anomaly’s existence, such as an operational model, Markov model, outlier model, clustering model, multivariate model, and time series model, etc. [44]. Knowledge-based anomaly detection uses a set of rules to identify malicious behavior; these rules are defined based on suspicious behavior observed from past knowledge of adverse attacks [45]. Therefore, it is also known as a rule-based anomaly detection technique. Machine learning-based anomaly detection uses the automatic approach of classifying normal and abnormal data with the help of a data mining approach [46].

#### 2.2.2. Classification of Anomaly Detection

Machine learning-based anomaly detection is classified into three main approaches, supervised, semi-supervised, and unsupervised anomaly detection. In recent research, a combination of these approaches is used in anomaly detection for NFV networks [47]. Researchers have proposed a method using semi-supervised learning to identify network anomalies and then using supervised learning to classify them as benign or malicious. Others have proposed using unsupervised learning for anomaly detection and then applying semi-supervised learning to identify the root cause of the anomaly. These approaches have shown promising results in detecting and mitigating anomalous behavior in NFV networks [48].

a. Supervised Model

In supervised anomaly detection, we create a model that works on a trained dataset and categorizes the data into two labels, i.e., normal and abnormal [49]. The system collects information regarding the network and compares it to the labeled data; if the data record is more likely to be routine data, then it is considered normal, while on the other hand, if the data are more likely to be abnormal, then it is considered to be an anomaly [50].

b. Semi-supervised Model

The supervised model depends on the labeled dataset; therefore, the labeled dataset should be of good quality [51]. A semi-supervised model works only on a single label, i.e., a normal dataset; in this approach, if the collected data do not match the normal dataset, then it is considered an anomaly, but this approach does not identify all types of anomalies [52].

c. Unsupervised Model

Unsupervised is an efficient but complicated approach to finding an anomaly in the network. It does not use any label dataset; it works on instance data and efficiently identifies novel anomalies in the network [53]. The unsupervised approach uses raw measurements and data related to normal behavior to help the system identify novel and dynamic anomalies. Therefore, it is also known as a behavior-based model. There are several unsupervised techniques, such as adaptive threshold-based, clustering, Bayesian belief networks, and principal component analysis [54].

#### 2.2.3. Causes of Network Anomaly Detection

**Figure 3:**
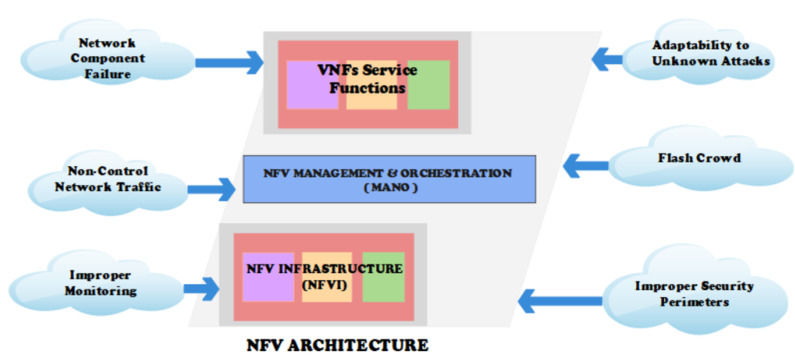
Causes network.

Anomaly detection identifies security vulnerabilities by finding anomalies in the system’s normal behavior [55]. There are several causes of network anomalies, such as network component failure, non-control network traffic, improper monitoring, improper security perimeters, flash crowd, etc. Figure 3 [56].

a. Network Component Failure

The network component includes hardware- and software-related components, such as routers, firewalls, VNFs services functions, etc. If these components fail during critical data communication, it causes an anomaly, a performance-related issue [57].

b. Non-Control Network Traffic

Non-control network traffic is a serious issue that causes the network to be unavailable; an attacker exploits this vulnerability and makes the victim server unavailable for legitimate users, which causes anomalies in the network traffic behavior [58].

c. Improper Monitoring

The access and login of an unauthorized user, weak security monitoring, avoiding unnoticed events, and interruption in the network all come under improper monitoring and cause anomalies in the network [59].

d. Improper Security Perimeters

Security perimeters include the security measures taken by the network administrator, and these perimeters also cause anomalies in the network. If security perimeters are not strong enough, the network will easily be compromised [60]. The attacker always tries to take advantage of such security vulnerabilities.

e. Flash Crowd

The flash crowd is also one of the causes of an anomaly in the NFV [61]. Flash crowd means the network is overloaded with legitimate traffic, and many legal users try to access the server, creating abnormal network traffic [62].

#### 2.2.4. Use Cases of Anomaly Detection

Anomaly detection is used in various scenarios in NFV networks to identify performance-related and security-related issues [63]. Some popular use cases of anomaly detection are intrusion detection, fraud detection, malware detection, data loss prevention, log anomaly detection, etc. [64]. The anomaly detection identifies anomalies in the NFV network in an automated way and generates alerts that help the network to take immediate countermeasures.

#### 2.2.5. Challenges of Anomaly Detection

Anomaly detection is a helpful technique for identifying unusual behavior, through which we detect existing and novel intruders or malicious attacks, and it also helps improve the performance of the NFV network [65]. Despite all these, there are several limitations of the anomaly detection technique, a few of which are discussed here.

(a) Runtime Anomaly Detection

Fast and reliable communication has always been the goal of NFV; we always want a solution that helps to identify runtime anomalies accurately by inspecting the data traffic without disturbing the legitimate traffic [66].

(b) Reducing False Alarm

The differentiation between normal and abnormal behavior is a difficult task; there is a chance that an anomalous event may occur close to normal behavior, and a normal event may occur close to anomalous behavior [67]. In both cases, we have a false alarm. Design such anomaly detection techniques that reduce this false alarm in NFV.

(c) Dimensionality Reduction

The appropriate selection of network traffic features is an important challenge in NFV. Select those network features for anomaly detection to help identify anomalous data traffic without compromising performance [68].

(d) Adaptability to Unknown Attacks

As the communication world grows, new challenges in the form of anomalies exist, which should be dynamically identified by anomaly detection techniques [69].

(e) Infrastructure Attacks

NFV is a virtual network environment that involves third-party to provide network infrastructure; several vulnerabilities exist in this network environment [70]. Therefore, an efficient anomaly detection system is required for such types of vulnerabilities.

Due to these issues, detecting anomalies in the NFV network is not easy. Researchers proposed several anomaly detection methods to overcome these limitations [48]. In this paper, we analyze some research paper that covers anomaly detection in NFV and discuss some of its limitations in Section 3.

## 3. Review and Comparative Analysis of State-of-the-Art Anomaly Detection in NFV

Recently, the detection of malicious attacks in the NFV network has received considerable attention, and new algorithms for detecting such attacks have been developed that use the anomaly detection technique. Anomaly detection can identify malicious attacks in the overall network, while the firewall detects malicious attacks only in the data that pass through the firewall [71]. Therefore, new algorithms for anomaly detection need to be developed to identify anomalies in the NFV network that should overcome all the limitations as we discussed in the previous section.

### 3.1. State-of-the-Art Anomaly Detection in NFV

In this section, we analyze some research papers that cover anomaly detection in NFV, focusing on the most recent and efficient machine learning algorithms proposed for detecting network anomalies.

#### 3.1.1. Anomaly Detection Using SMNRT

Derstepanians, Arman, et al. (2022) [66] proposed a machine learning-based approach for detecting anomalies in network function virtualization (NFV) infrastructures. The proposed method, simple median near real-time (SMNRT), is a hybrid approach that combines unsupervised and supervised learning techniques. The unsupervised part of the system uses a clustering algorithm to group similar data points into clusters, with anomalous data points identified as outliers. The supervised part trains a machine learning model to classify data points as either normal or anomalous. The proposed system is evaluated on a dataset of network traffic data from a real-world NFV infrastructure and achieves high detection accuracy, with an F1 score of over 0.9. The paper’s methodology involves four main steps, including data pre-processing, feature extraction, unsupervised clustering, and supervised classification. The evaluation of the proposed system includes comparing its detection performance with other state-of-the-art anomaly detection methods, demonstrating its effectiveness in detecting anomalies in near real time with high accuracy.

#### 3.1.2. Matrix Differential Decomposition

Chen, Jing, et al. [72] proposed the matrix differential decomposition (MDD) method of anomaly identification in the NFV network. They designed a technique that works in three phases. In the first phase, a prototype model is implemented in the NFV network that collects and monitors the NFV network traffic, and its behavior is analyzed. The second phase implements the matrix differential decomposition model (MDD) that identifies the anomaly in the NFV network. In the last step, the proposed algorithm is tested experimentally, evaluated on three NFV networks individually, and its outcomes are studied. The MDD algorithm for anomaly detection and localization not only gives good results in identifying multiple anomalies at a single time but also prevents anomalies due to the localization of network devices.

#### 3.1.3. Machine Learning-Base Early Anomaly Detection

Elmajed, Arij, Armen Aghasaryan, and Eric Fabre et al. [73] presented a machine learning-based anomaly detection algorithm focusing on two main challenges to identifying the anomaly in the NFV network: first, to detect faults before they severely affect the network, and secondly, to take countermeasures before the unavailability of NFV services. For this purpose, an experimental cloud-based NFV application was created that is isolated from all other applications, and this environment contains few virtualized network functions. The authors injected a series of resource perturbations and collected multiple metrics of the system behavior. In the next step, using different machine language approaches, they identified the anomaly in the system. They studied four machine learning (ML) approaches and compared their metrics results; the random forest (RF), XGBOOST, and KNN algorithms gave accuracy above 90%, while the max-likelihood classifier had 84% accuracy. After analyzing the fault localization and identification performance, RF and XGBOOST gave the best results in classifying the different types of anomalies. Despite these results, the proposed model further needs to improve the method of anomalies in the NFV network in a more generalized way.

#### 3.1.4. Tree-Based Anomaly Detection

Girish, L., et al. [74] discussed the isolation forest algorithm for anomaly detection in NFV networks, which is an unsupervised anomaly detection approach. In this method, each occurring event of the data can be efficiently separated and works as a decision tree. The highly sensitive nature of the isolation forest helps to isolate abnormal data points toward the end nodes of the decision tree and normal data points toward the root. The feature that kept anomalies isolated from normal points originally helps to detect abnormalities in the NFV network. The isolation forest algorithm is tested by injecting the anomalies in the NFV network and collecting 12 different metrics’ data. Results show that the isolation tree algorithm efficiently detects anomalies dynamically in the NFV network.

#### 3.1.5. SLA-Aware Anomaly Detection

Hong, Jibum, et al. [75] proposed a machine learning algorithm for anomaly identification in NFV networks using service level agreement (SLA) violation and some of the VNF performance features. The SLA-Aware algorithm work in three steps. The first step is virtual network orchestration, in which a monitoring function operates on NFVI (NFV infrastructure) and collects data on different VNFs in the network in terms of metrics. The second preprocessing step converts the collected information into valid training models and analyzes the data regarding anomaly detection. They divided the data into two categories; normal and abnormal data. The anomalous data are further categorized based on VNF performance and SLA violations. VNF performance includes data that identify packet drops due to the unavailability of VNF resources. SLA violations contain data representing the time that the service does not respond to the request. The last step is training models; in this step, among several machine learning-supervised anomaly detection algorithms, they selected the four best models based on testing. The chosen algorithms are distributed Ran.F (random forest), Gradient Bo.M (boosting machine), Extreme G_B (X gradient boost), and Feed_forward NN (F neural network). The Gradient Bo.M algorithm performs best among these four top algorithms. The results show that the implemented architecture of 95% accurately identified anomalies in the NFV network.

#### 3.1.6. Markov Chain and K-Means Method

Blaise, Agathe, Stan Wong, and A. Hamid Aghvami, et al. [76] proposed a decision-based machine learning algorithm to identify the anomaly in the NFV network. They analyzed the VNFs service in a forward and backward sequence and found the normal and abnormal patterns of network functions. On detecting any anomaly, an alert is generated and a message is sent to the administrator to isolate the NFV network. The whole method is divided into two parts. The first part analyzes the virtual network function services using the Markov chain algorithm. In contrast, in the second part, the K-mean pattern detection technique is used to distinguish the normal behavior or abnormal behavior of the NFV services. The property of the system’s future state depends on the current state because Markov does not store any information; therefore, it is free from history. We apply the properties of the Markov chain function both forward and backward. Every VNF represents the state in terms of two transition metrics that also show their connection. The K-mean creates data clusters and uses them to analyze the network behavior. Since K-Mean works on clustered data, it identifies anomalies more accurately than other algorithms and can produce more accurate results as the cluster size increases.

#### 3.1.7. Distance-Based Anomaly Detection in NFV

In Ref. [77], the proposed framework designs a legitimate behavior model at runtime to monitor the network traffic in an NFV network. When an anomaly is detected, the administrator initiates a mitigation process using the root cause analysis technique. This method uses distance-based clustering techniques to develop a legitimate model for anomaly detection. The proposed method efficiently identifies anomalies with low latency rates and reduces the false alarm rate.

#### 3.1.8. Intelligent Orchestration of NFV for Anomaly

Silva, Fernando, and Alberto Schaeffer-Filho, et al. [78] proposed a method for anomaly detection in NFV using a supervised learning technique. The method was implemented in NFV orchestration and management block to monitor the data traffic. The main objective of this technique is to monitor all the incoming traffic; if any anomaly or malicious traffic is found, the proposed module automatically instantiates the network function, which helps with anomaly mitigation. The proposed method is efficient because it is integrated with the NVF orchestration and management module and minimizes resource usage. The experimental evaluation shows that the proposed method identifies anomalies with more than 90% accuracy in the NFV network.

#### 3.1.9. IFTM-Based Anomaly Detection in NFV

Schmidt, Florian, et al. [79] proposed a model that implemented an unsupervised learning approach for anomaly detection in the NFV network. This method consists of an automatic function for identification and a threshold-based technique for classifying data traffic into normal and abnormal behavior. Due to two main tasks, i.e., identity and threshold values, the proposed method is called IFTM. IFTM identifies anomalies dynamically. The first function monitors the network traffic and identifies its behavior; if the traffic data are found to be abnormal, they are sent to the threshold function, where we classify their behavior as malicious or normal. This method gives 98% accurate results and also reduces the false alarm rate. However, this method has some limitations; the IFTM method is an expert system and needs some administrative control to handle it. Therefore, a method should be designed with an automatic system for anomaly detection without the intervention of any administration.

#### 3.1.10. LSTM-Based Anomaly Detection in NFV

Alessio Diamanti, Jose Manuel. S.V., et al. [80] proposed an event-driven unsupervised machine-learning method to detect anomalies in the NFV network. This method provides a fully automated anomaly detection solution and identifies anomalies at runtime. The proposed method works in two steps; in the first step, they designed different software modules for other network functions. They used long short-term memory (LSTM) autoencoders and identified whether the data were nominal or anomalous. In the second phase, if any anomaly was found, it was sent to the root cause analysis module, where the anomaly’s mitigation occurs. The LSTM autoencoder works on the radiography visualization approach. This method identifies anomalies dynamically in a heterogeneous environment. This method gave 90% accurate results, but this method works on the virtual layer. Therefore, the proposed design should be extended to physical and cross-layer anomalies.

#### 3.1.11. Unsupervised Neural Network SOM

Lanciano, Giacomo, et al. [81] presented an approach for detecting anomalies in virtual networks using unsupervised machine learning techniques. The proposed method involves the use of a self-organizing map (SOM), which is an unsupervised neural network that can cluster similar data points together. The SOM is trained on network traffic data to create a map of the normal network behavior. During the detection phase, new traffic data are input into the SOM, and if the data deviate significantly from the normal behavior, an anomaly is detected. The authors evaluated their approach on a simulated virtual network environment and found that it was able to detect various types of anomalies, including denial-of-service (DoS) attacks and port scans, with a high degree of accuracy. Overall, the paper demonstrates that unsupervised machine learning techniques can be effective for detecting anomalies in virtual networks, and the proposed SOM-based approach shows promise for this task.

### 3.2. Comparative Analysis of State-of-the-Art Anomaly Detection in NFV

All the above-proposed methods for anomaly detection in the NFV have some strengths and weaknesses. The method proposed by Derstepanians, Arman, et al. [66] was useful for telecommunication and infrastructure services. Telecommunication providers are always ready to deploy easily configurable and cost-effective solutions. Derstepanians, Arman, et al. [66] therefore designed a model that is easily implemented within the VNF services module and uses both supervised and unsupervised methods for anomaly detection. Their approach used VM data for anomaly detection. The method proposed by Jing Chen [72] is a matrix decomposition method, in which they use a three-step procedure to detect the anomaly and solve the device localization problem that generates the anomaly. This method not only gives good results but also reduces the presence of anomalies in the NFV network through the localization of devices.

Arij Elmajed [73] proposed a runtime solution for anomaly detection and focused on two main tasks: detecting anomalies before they affect system performance and taking timely countermeasures. Arij Elmajed [73] implemented his method using four different machine learning algorithms and studied their behavior in terms of accuracy. Girish and Dr. Sridhar [74] used the isolation forest algorithm technique to identify anomalies in the NFV network and create a decision tree for data. This decision tree dynamically separates the anomalous data from the norm data and shows good results. Jibum Hong, Suhyun, and Jae Hyoung [75] used service level agreement (SLA) and performance characteristics of VNF. This approach works in three steps: monitoring data traffic, analyzing data, and taking countermeasures to mitigate anomalies. The analyzed phase plays a major role in detecting anomalies. It separate the data into two main categories: anomalies due to VNF performance or SLA violations.

Agathe Blaise [76] proposed a decision-based machine algorithm that detects anomalies in forward and backward sequences and generates an alert message. The method works in two steps. The first uses the Markov chain algorithm to detect anomalies, and the second uses the K-means algorithm to generate an alert message if any anomaly occurs.

Anton Gulenko, Florian Schmidt [79] used a distance-based clustering model to identify anomalies in a NFV network and implement a mitigation process using root cause analysis. This method relies on human interaction to deal with anomalies but has low latency and reduces the false alarm rate. Fernando Silva and Alberto, Schaeffer-Filho [78] proposed a method implemented in the NFV orchestration and management block to identify anomalies in the network without any human interaction automatically. The proposed method is efficient and reduces resource consumption. Florian Schmidt and Anton Gulenko [77] proposed a method that automatically identifies anomalies in the network’s data traffic and, after finding any anomalous data, checks whether the data are malicious or not. For this check, it uses a threshold value. Alessio Diamanti, Jose Manuel [80] proposed a method that identifies anomalies in a heterogeneous environment and provides zero-touch network orchestration in the NFV. This method dynamically and automatically detects anomalies but operates at the virtual layer of the NFV network [82]. Here, we compare these methods and analyze their efficiency and effectiveness in identifying anomalies in NFV. Table 2 presents the overall analysis of the review papers. From the above-proposed methods, we conclude some important facts regarding the anomaly detection technique in the NFV:

- Supervised methods identify anomalies in the NFV network more quickly and accurately as compared to unsupervised methods.
- Supervised methods are either implemented in NFV orchestration and management block or VNFs services function block; this technique reduces the cost and resource utilization.
- Unsupervised methods are complex compared to supervised methods but detect novel anomalies in the NFV network.
- Unsupervised methods provide a runtime anomaly detection mechanism and are implemented as separate modules or service functions.
- Unsupervised methods have more false alarm rates than supervised methods [83].
- Unsupervised methods provide a more generalized solution for anomaly detection than supervised methods.
- Supervised methods also provide a mitigation process using root cause analysis and reduce costs by integrating with the NFV infrastructure.
- Unsupervised methods provide a zero-touch network [80] monitoring environment and automatic anomaly detection approach in the NFV, whereas supervised methods need human interaction to handle anomalies.
- Unsupervised methods also work in heterogeneous data environments in runtime scenarios [79].

We also study other surveyed papers on state-of-the-art anomaly detection in the NFV network. For instance, Wang, Song, et al. [22] (2021) discuss anomaly detection in network security, including various machine-learning techniques that can be used for anomaly detection. However, they do not specifically focus on anomaly detection in NFV-based networks. Nonetheless, the paper provides a good overview of the techniques that can be used for network anomaly detection, which could be useful in the context of NFV-based networks as well [84]. A summary of the surveyed papers and their methodologies is provided in Table 2. The table presents an overview of the different papers, highlighting their key contributions and the methodologies they employed for anomaly detection in NFV networks. This summary will help in comparing and understanding the approaches taken by each paper and their relevance to our own survey.

**Table 2:** Comparison of different anomaly detection methods.

Proposed Methods	Year of Publication Strengths	Anomaly Detection Approach	ML-Based Algorithm	Strengths	Limitations	Accuracy	Future Work
SMNRT [66]	2022	supervised	HYBRID MODEL	Suitable for time-sensitive applications, high detection accuracy. Hybrid techniques detect anomalies in a complex and dynamic network.	Relies on labeled data for supervised learning. System’s performance may be affected by the quality. The system may require significant computational resources and expertise in machine learning.	98%	More ML algorithms and neural network models. Address the issue of false positives. Improve real-time performance. Evaluate the proposed system in a real-world setting.
MDD [72]	2019	unsupervised	PCA	Multiple anomaly detection, handle localization, reduces computation and deployment difficulties.	Dynamic anomaly detection is not possible.	97.24%	Develop an online-based anomaly detection system.
MLBEAD [73]	2020	supervised	Ran.F	Early detection of anomalies, anomaly handling mechanism, online detection.	A limited no. of anomalies are identifiable and need generalization, multiple algorithms are used.	93%	Develop a more generalized mechanism for anomaly detection and also implement it in the Docker platform.
TBAD [74]	2019	unsupervised	Dec.T	Efficient anomaly detection, strong defense mechanism, timely detection of anomaly.	A small-level, real-time implementation required.	90%	Develop a real-time online anomaly detection mechanism and use the same method with deep learning techniques.
SAAD [75]	2020	supervised	G.Bo.M	Strong identification for anomalies, good for web hosting scenarios.	Work only for web hosting, required generalization.	95%	Extend the proposed method for large VNF scenarios.
MCKM [76]	2018	unsupervised	MC	Easily and more accurately identifies the anomaly in NFV, good defense mechanism, suitable for a large network, scalable.	Computational and resource utilization overhead.	85%	Develop the same model using a deep-learning algorithm.
DBCAD [77]	2018	Unsupervised	DBCA	Strong defense mechanism against anomalies. Runtime legitimate behavior model for anomaly detection, low latency rate.	Detection of particular types of anomalies, more resource utilization. Human interaction required for mitigation.	98.9%	Develop a more generalized model that covers all types of anomalies and automatically handles all processes from detection to mitigation.
IOCNF [78]	2022	Supervised	Fuzzy Logic	Minimize resource usage. Automatically mitigate anomalies in NFV. Work together with network orchestration and management module.	Work only limited datasets. Fewer features are considered for anomaly detection.	90%	The method should be generalized. Consider large data traffic features.
IFTM [79]	2018	Unsupervised	LSTM	Reduces false alarm rate. Use the expert system to identify anomalies.	Depend upon administrative control. The method covers some specific data traffic.	98%	Design should extend to the automatic detection of anomalies in NFV. The method should be generalized.
SYRROCA [80]	2020	Unsupervised	LSTM	Detect anomalies in a heterogeneous environment. Identifies anomaly dynamically and automatically. Provides zero-touch network orchestration in NFV.	Work only at the virtual layer. Use large metrics for anomaly detection.	98%	Design should extend to physical and cross layers. Design for other types of data streams, such as VoIP, 4G, 5G, etc.
SOM [81]	2021	Unsupervised	Clustering	more accurate and efficient results. Joint analysis of system-level and application-level metrics. Effective in identifying similar input patterns.	Number of hyper-parameters that have to be decided. Non-negligible processing time. Not suitable for excessively large networks.	99%	Hyperparameters to improve its accuracy and reduce false positives. Exploration of other unsupervised machine learning techniques, such as clustering.

### 3.3. Quantitative Comparison of State-of-the-Art Anomaly Detection in NFV Network

In the context of this survey on anomaly detection in NFV using machine learning, this subsection undertakes a detailed analysis of anomaly detection by evaluating the parameters extracted from Section 2 of the literature. A comparison of cutting-edge anomaly detection solutions is provided in Table 3, offering an overview of their key features and a quantitative comparison of different proposed methods for anomaly detection in NFV networks using supervised and unsupervised learning. The performance of each method is evaluated based on four metrics: accuracy, precision, recall, and F1-score. These metrics help to determine which solution offers the most accurate and effective detection of anomalous events in the network.

Accuracy measures how often a model correctly predicts the outcome. Precision measures how often the model is correct when it predicts a positive outcome. It is like asking “How many of the positive predictions were correct?” Precision is important when we want to avoid false alarms. Recall measures how often the model correctly predicts a positive outcome out of all the true positive outcomes. Recall is important when we want to ensure that we do not miss any positive outcomes. The F-measure is a harmonic mean of precision and recall. The F-measure is useful when we want to balance the trade-off between precision and recall.

**Table 3:** Quantitative comparison of different anomaly detection methods.

Proposed	Accuracy	Precision	Recall	F1-Score
SMNRT [66]	0.98	0.83	0.98	0.90
MDD [72]	0.9724	0.89	0.91	0.90
MLBEAD [73]	0.93	0.92	0.89	0.91
TBAD [74]	0.90	0.92	0.91	0.92
SAAD [75]	0.95	0.94	0.89	0.91
MCKM [76]	0.85	0.95	0.95	0.95
DBCAD [77]	0.989	NSp	NSp	NSp
IOCNF [78]	0.90	0.91	0.92	0.91
IFTM [79]	0.98	NSp	NSp	NSp
SYRROCA [80]	0.98	0.94	0.93	0.94
SOM [81]	0.9959	0.9803	0.9992	0.9896

Through the comparison of accuracy, recall, precision, and F1-score across different anomaly detection methods, researchers can determine which method is the most effective in detecting anomalies in the NFV network. It is essential to focus on these quantitative features to ensure that the selected method can accurately and effectively identify anomalies while minimizing false positives. The metrics are presented in a tabular form for each proposed method, except for DBCAD and IFTM, which are marked as “NSP” (not specified) due to the lack of reported results in the corresponding paper. The table aims to provide a quick comparison of the performance of different proposed methods in terms of anomaly detection accuracy. The accuracy metric indicates the percentage of correctly classified instances among all instances. According to this metric, SOM [81] shows the best performance among all methods, achieving an accuracy of 0.9959, followed by SMNRT [66] and SYRROCA [80] with accuracies of 0.981 and 0.974, respectively.

Precision indicates the proportion of true positives to the total number of positive predictions. Among all methods, SOM [81] shows the best precision with a score of 0.9803, followed by MCKM [76] with a precision score of 0.95.

Recall indicates the proportion of true positives to the total number of actual positive instances. Among all methods, SOM [81] shows the highest recall score with a value of 0.9992, indicating that it can identify almost all positive instances as anomalies.

Among all methods, SOM [81] also shows the highest F1-score with a value of 0.9896, followed by SMNRT [66] and SYRROCA [80] with F1-scores of 0.976 and 0.94, respectively.

Overall, the results suggest that SOM is the most effective method for anomaly detection in NFV networks based on the considered performance metrics. However, it is important to consider other factors, such as complexity, scalability, and robustness, when choosing an appropriate anomaly detection method for a specific NFV environment.

**Figure 4:**
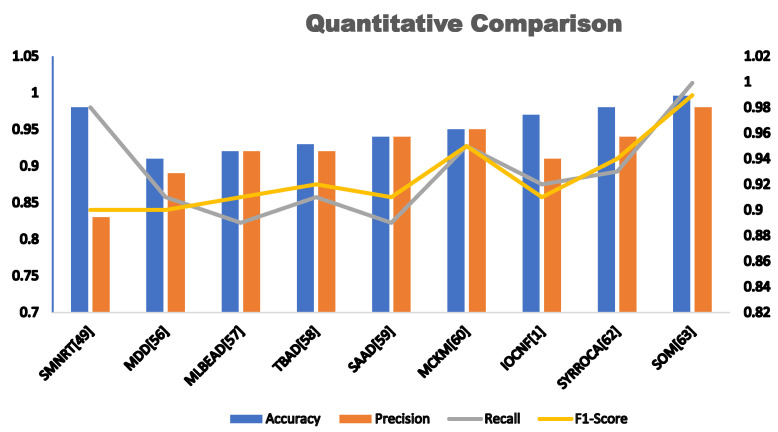
Graphical representation of the quantitative comparison between different anomaly detection methods.

The following graph, as shown in Figure 4, provides a graphical representation of the quantitative comparison between different anomaly detection methods, which better represents the variations in accuracy, precision, recall, and F1 scores.

## 4. Open Research Issues and Challenges

The field of network-based anomaly detection in NFV networks using supervised and unsupervised approaches of machine learning is still evolving, and several areas of research remain open. One such area is developing methods that can automatically and accurately detect anomalies in the NFV network. These methods should be capable of identifying the source of the attack without compromising NFV functionality and quality of service. Additionally, these methods should be able to take timely and effective countermeasures and identify new anomalies while reducing false alarms.

Another area that requires further research is the development of an effective anomaly detection mechanism that can overcome the vulnerabilities of the shared environment in NFV networks. The mechanism should be designed to ensure that the anomalies are detected in real time and that they do not impact the performance of the network [85].

Moreover, there is a need to explore the identification of new and advanced machine-learning techniques that can be used for anomaly detection in NFV networks. This could involve combining different techniques and developing hybrid models to improve the accuracy and efficiency of anomaly detection.

**Figure 5:**
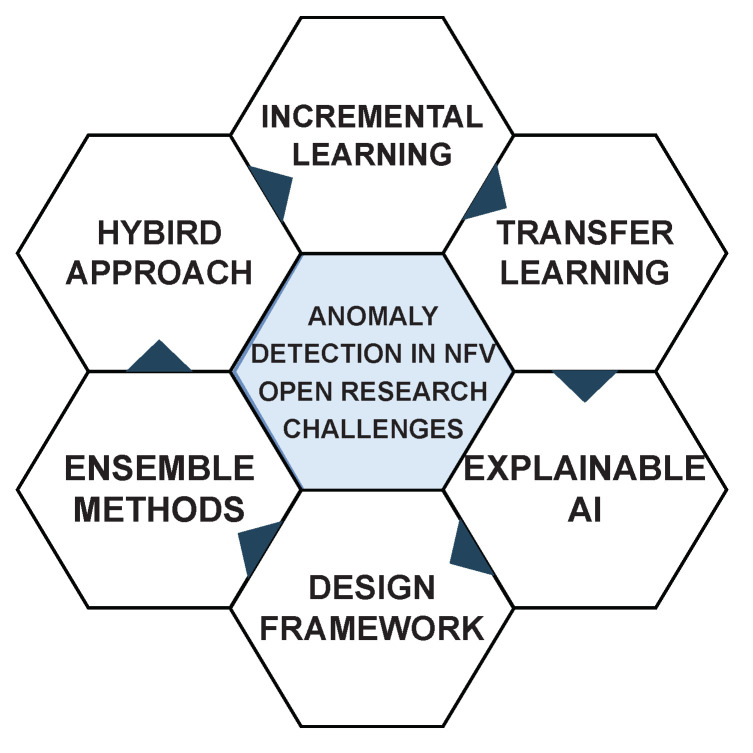
Open research challenges of anomaly detection in NFV.

Finally, there is a need to identify new security issues and challenges in each environment and develop new techniques and strategies to address them effectively. This would require constant research and development to keep up with the rapidly evolving threats in the NFV network. However, the area of network-based anomaly detection in NFV networks using supervised and unsupervised approaches of machine learning is an active research area, and many opportunities for research and innovation still exist, a few of which are illustrated in Figure 5 discussed below.

### 4.1. Hybrid Approaches

Hybrid approaches that combine supervised and unsupervised machine learning techniques offer a promising direction for research in anomaly detection in NFV networks. By leveraging the strengths of multiple techniques, these approaches have the capability to surmount the constraints of individual methods and offer enhanced accuracy and efficiency in detecting anomalies in the complex NFV environment [86]. Supervised learning approaches rely on labeled data to train a model to identify anomalies. However, labeled data are often scarce in NFV environments, which limits the effectiveness of supervised approaches. Unsupervised approaches, while not relying on labeled data, may have difficulty detecting unprecedented or advanced attacks.

By combining the advantages of supervised and unsupervised approaches, hybrid methods can offer more efficient anomaly detection [87]. One such hybrid approach is to use unsupervised techniques for detecting potential anomalies, and then supervised techniques for classifying them as normal or malicious. Another approach could entail using unsupervised techniques to establish normal patterns and then utilizing supervised methods to identify any deviations from those patterns [88].

Research in hybrid approaches could also explore the use of deep learning techniques, such as deep autoencoders, which can learn representations of data without the need for explicit feature engineering [89]. Deep learning techniques have shown promise in other domains and could be adapted for use in anomaly detection in NFV networks.

Overall, research in hybrid approaches offers an exciting direction for advancing the field of anomaly detection in NFV networks. By combining the strengths of different techniques, hybrid approaches could provide more accurate and efficient detection of anomalies, while reducing false positives and false negatives.

### 4.2. Incremental Learning

Incremental learning is a machine learning approach that enables an anomaly detection system to continuously learn and improve its detection capabilities over time. This is particularly important in NFV networks, which are dynamic and constantly evolving, and where new types of attacks and anomalies may arise [90].

By using incremental learning techniques, an anomaly detection system can adapt to changes in the NFV network and learn from new data in real time. This allows the system to detect and identify previously unknown anomalies and attacks, and to improve its overall accuracy and effectiveness [91].

There are several challenges involved in implementing incremental learning in anomaly detection systems for NFV networks. One of the key challenges is the need to handle large volumes of data, as well as the need to process data in real time. To address these challenges, researchers have developed specialized algorithms and techniques, such as online learning and stream mining, that can process data in real time and adapt to changes in the network [92].

Another challenge is the need to balance accuracy and efficiency. Incremental learning algorithms may require significant computational resources and may be computationally expensive. Therefore, it is important to develop efficient algorithms that can detect anomalies accurately while minimizing false positives and reducing the risk of false alarms [93].

Despite these challenges, incremental learning represents a promising area of research for improving anomaly detection in NFV networks. By continuously learning and adapting to changes in the network, anomaly detection systems can improve their effectiveness and better protect the network against new and emerging threats.

### 4.3. Transfer Learning

Transfer learning is a powerful machine learning approach that involves transferring knowledge from a sender domain to a destination domain. In the realm of anomaly detection in NFV networks, transfer learning can be applied to exploit knowledge acquired from one network and apply it to another network with similar characteristics, thereby enhancing the performance of the anomaly detection system [94].

In NFV networks, transfer learning can be useful in situations where there are multiple networks that have similar characteristics but with different traffic patterns and behavior. By transferring knowledge from one network to another, the anomaly detection system can learn to identify anomalies in the new network more effectively and efficiently [95]. For example, if an anomaly detection system is trained on a network with similar traffic patterns and characteristics as the target network, it may be able to identify new and emerging anomalies in the target network more quickly and accurately.

Transfer learning can reduce the amount of labeled data needed for training an anomaly detection system in the target domain and can mitigate the problem of dataset shift. In NFV networks, transfer learning can be applied through inductive, transductive, and multi-task learning approaches. Inductive transfer learning is useful when labeled data are limited, while transductive transfer learning is suitable when there is a significant amount of unlabeled data available. Multi-task learning can be effective for detecting anomalies in multiple networks with similar characteristics [96].

Overall, transfer learning is a promising research area for improving the performance of anomaly detection systems in NFV networks. By leveraging knowledge from one network to another, transfer learning can help to address the challenges of limited labeled data and dataset shift, and improve the accuracy and efficiency of anomaly detection in NFV networks [97].

### 4.4. Ensemble Methods

Ensemble techniques refer to the combination of multiple anomaly detection algorithms to enhance the overall performance of the system. The idea behind ensemble methods is that by combining the outputs of several individual models, the overall accuracy and robustness of the system can be improved. This approach can be particularly effective when the individual models have different strengths and weaknesses, as the weaknesses of one model can be offset by the strengths of another [98].

In the context of anomaly detection in NFV networks, ensemble methods can be used to reduce false positive rates and improve the accuracy of the detection system. By combining multiple algorithms, the system can more accurately detect anomalies and reduce the likelihood of detecting false positives [99].

Ensemble methods, such as bagging, boosting, and stacking, can be used for anomaly detection. Bagging involves training multiple models on different subsets of data, while boosting trains models sequentially to focus on misclassified samples. Stacking combines the outputs of multiple models to create a final prediction. These methods are useful when individual models have high variance and bias, or complementary strengths and weaknesses [100]. In the context of anomaly detection in NFV networks, there is potential for ensemble methods to improve the accuracy and robustness of existing systems. Additional research is required to investigate the efficiency of diverse ensemble methods and to create novel techniques that are customized to the particular traits of NFV networks.

### 4.5. Explainable AI

Explainable AI (XAI) is a research area that aims to develop machine learning models that can provide understandable explanations for their decisions or predictions. In the context of anomaly detection in NFV networks, XAI can be used to make the detection process more transparent and interpretable for network administrators [101]. This can help network administrators to better understand the detection results and make informed decisions about security measures.

One of the challenges of anomaly detection in NFV networks is the high volume and complexity of data generated by the network. Machine learning models are often used to process these data and detect anomalies. However, these models can be difficult to interpret, especially when they use complex algorithms, such as deep neural networks [34]. This can make it difficult for network administrators to understand how the models arrived at their decisions, which can lead to mistrust of the detection system and difficulty in making informed decisions [102].

XAI techniques can be used to address this challenge by providing interpretable explanations for the decisions made by the machine learning models [103]. For example, decision trees can be used to create rules that explain how a model arrived at a decision. Alternatively, visualization techniques can be used to create graphs or heat maps that show which features were most important in the decision-making process [104].

XAI can also be used to provide context for the anomalies detected in the network. For example, if a model detects an anomaly in a particular network function, XAI techniques can be used to explain why that function is critical to the overall performance of the network and what the potential consequences of a failure in that function might be [105]. This can help network administrators prioritize their responses to anomalies and take appropriate actions to mitigate the risk.

Overall, XAI is an important research area in anomaly detection in NFV networks, as it can help to improve the transparency and interpretability of machine learning models, which can in turn improve the trust and effectiveness of the detection system.

### 4.6. Design Framework

The development of a design framework is an essential area of research for improving anomaly detection in NFV networks. The NFV architecture includes multiple layers of hardware and software, and each layer has its functionalities and requirements [17]. A design framework can help in the development of an effective and efficient anomaly detection system that can operate in all layers of the NFV network.

The framework should take into account the characteristics of each layer and develop a methodology to detect anomalies in each of them. For example, the hardware layer may have its own set of vulnerabilities that can be exploited by attackers. The framework should provide a set of techniques and tools to detect such anomalies [106].

Similarly, the virtual functional layer may have a different set of vulnerabilities that require a different set of tools and techniques for anomaly detection. The framework should take into account these differences and develop a set of strategies that can be used to detect anomalies in the virtual functional layer [107].

Finally, the hardware–virtual functional layer is a combination of both the hardware and virtual functional layers. The framework should be capable of detecting anomalies in this layer by combining the techniques and strategies developed for the hardware and virtual functional layers [75].

The design framework can help to develop a comprehensive approach to detect anomalies in the NFV network. It can provide a systematic way to integrate various anomaly detection techniques and strategies to detect anomalies effectively and efficiently [108]. The framework can help to reduce false positives and false negatives, which are major challenges in anomaly detection. It can also help network administrators quickly identify the source of the anomalies and take timely and effective countermeasures to protect the NFV network from attacks.

## 5. Conclusions and Future Work

Network function virtualization (NFV) is a modern concept prevalent in cloud environments. NFV simplifies deployment and management while offering greater flexibility, scalability, and lower network infrastructure costs. It is transforming traditional hardware-based networks into software-driven networks. Despite these benefits, a significant drawback cannot be ignored: security vulnerabilities and cyber attacks. As NFV is a shared-based software system, it relies on a third-party infrastructure, making it an attractive target for attackers. Anomaly detection is an effective technique for the identification and prevention of cyber attacks. It is a broad field that helps identify different types of network traffic-based security attacks. This paper focuses on network traffic-related security issues and analyzes different anomaly detection methods in the NFV network. Our research shows that several effective machine learning algorithms exist, which we divide into supervised and unsupervised categories. We draw a few conclusions from the analyzed algorithms for anomaly detection in NFV:

- The unsupervised algorithm works efficiently on cluster data, while the supervised algorithm first trains the system and then implements the output results.
- Anomaly detection methods that work on the principle of unsupervised algorithms give less accurate results than supervised algorithms.
- Anomaly detection methods that use supervised algorithms could be generalized, which is not easier in unsupervised algorithms.
- Supervised algorithms are accurate and faster to implement than unsupervised algorithms.
- A separate module design is a better solution for anomaly detection in NFV networks.

This survey paper provides an updated and comprehensive review of the recent research on network-based anomaly detection in NFV networks using supervised and unsupervised machine learning approaches. Unlike previous surveys, this paper discusses the latest developments in anomaly detection mechanisms, hybrid models, and advanced machine learning techniques, and provides practical insights for improving the detection performance of NFV networks [109].

Moreover, this paper identifies the future research areas that require further investigation, including the development of effective anomaly detection mechanisms that can overcome the vulnerabilities of the shared environment in NFV networks, and the identification of new security issues and challenges. Additionally, this paper proposes some potential solutions, such as ensemble methods, incremental learning techniques, transfer learning, and explainable AI, to address these challenges.

Overall, this survey paper provides a more comprehensive and updated analysis of the state-of-the-art research on network-based anomaly detection in NFV networks, and it can serve as a valuable resource for researchers, practitioners, and network administrators working in this field.

## Figures and Tables

**Table 1:** Comparison of our survey with existing survey papers on anomaly detection in the NFV network using machine learning.

Survey Paper	Year	Structured Approach	Advantages/Limitations	Critical Assessment	Coverage of Other Techniques	Technical Difficulty	Performance Comparison
Our Survey	2023	✓	✓	✓	✓	✓	✓
Pang [20]	2021	✓	✓	✓	X	X	X
Nassif [21]	2021	✓	✓	✓	X	X	X
Wang [22]	2021	✓	✓	✓	X	✓	✓
Gebremariam [23]	2019	✓	✓	✓	X	X	X
Alam [24]	2020	✓	✓	✓	X	X	X
Ghaffar [25]	2021	✓	✓	✓	X	X	X
Lohrasbinasab [26]	2022	✓	✓	✓	X	X	X
Shah [27]	2022	✓	✓	✓	X	✓	✓
Gallego-Madrid [28]	2022	✓	✓	✓	X	X	X
Ahmed [29]	2021	✓	✓	✓	X	X	X
Nunez-Agurto [30]	2022	✓	✓	✓	X	X	X
Di Mauro [31]	2021	✓	✓	✓	X	✓	✓

## Data Availability

Not applicable.

## Abbreviations

The following abbreviations are used in this manuscript:

SMNRT	Simple Median Near Real-Time
MDD	Matrix Differential Decomposition
MLBEAD	Machine Learning-Based Early Anomaly Detection
TBAD	Tree-Based Anomaly Detection
SAAD	SLA-Aware Anomaly Detection
MCKM	Markov Chain and K-means Method
DBCAD	Distance-based Clustering Anomaly Detection
BBA	Black Box Approach
Ran.F	Random Forest
G.Bo.M	Gradient Boosting Machine
Dec.T	Decision Tree
MC	Markov Chain
DBCA	Distance-based Clustering Algorithm
PCA	Principle Component Analysis
SYRROCA	System Radiography and Root Cause Analysis
IFTM	Identity Function and Threshold Model
IOCNF	Intelligent Orchestration of Containerized Network Function
SOM	Self-Organizing Map
NSp	Not Specified
